# Routing Topologies of Wireless Sensor Networks for Health Monitoring of a Cultural Heritage Site

**DOI:** 10.3390/s16101732

**Published:** 2016-10-19

**Authors:** Sofía Aparicio, María I. Martínez-Garrido, Javier Ranz, Rafael Fort, Miguel Ángel G. Izquierdo

**Affiliations:** 1 Instituto de Tecnologías Físicas y de la Información “Leonardo Torres Quevedo” (ITEFI) CSIC, C/Serrano, 144, 28006 Madrid, Spain; javier.ranz@csic.es; 2 CEI Campus Moncloa, UCM-UPM y CSIC, 28040 Madrid, Spain; mi.martinez.garrido@csic.es (M.I.M.-G.); rafael.fort@csic.es (R.F.); miguelangel.garcia.izquierdo@upm.es (M.Á.G.I.); 3 Instituto de Geociencias, IGEO, (CSIC-UCM), C/José Antonio Novais, 2, 28040 Madrid, Spain; 4 E.T.S.I. Telecomunicación (UPM), Av. Complutense 30, 28040, Madrid, Spain

**Keywords:** wireless sensor networks, routing topologies, mesh topology, tree topology, cultural heritage

## Abstract

This paper provides a performance evaluation of tree and mesh routing topologies of wireless sensor networks (WSNs) in a cultural heritage site. The historical site selected was San Juan Bautista church in Talamanca de Jarama (Madrid, Spain). We report the preliminary analysis required to study the effects of heating in this historical location using WSNs to monitor the temperature and humidity conditions during periods of weeks. To test which routing topology was better for this kind of application, the WSNs were first deployed on the upper floor of the CAEND institute in Arganda del Rey simulating the church deployment, but in the former scenario there was no direct line of sight between the WSN elements. Two parameters were selected to evaluate the performance of the routing topologies of WSNs: the percentage of received messages and the lifetime of the wireless sensor network. To analyze in more detail which topology gave the best performance, other communication parameters were also measured. The tree topology used was the collection tree protocol and the mesh topology was the XMESH provided by MEMSIC (Andover, MA, USA). For the scenarios presented in this paper, it can be concluded that the tree topology lost fewer messages than the mesh topology.

## 1. Introduction

Wireless sensor networks (WSNs) belong to a new field of research that is currently undergoing rapid growth. A WSN is composed of nodes; each of them has computing power and can transmit and receive messages over wireless communication links. In recent years, WSNs have been used for structural health monitoring of different types of structures as bridges [1,2].

Over the last decade, there is a growing effort to protect and support cultural heritage. The need to protect our heritage from the environmental degradation is widely recognized by, e.g., the European Commission. Research has focused on solutions to preserve movable and immovable cultural heritage assets using WSNs [3,4,5,6,7,8,9,10]. This work belongs to a series of papers dedicated to study the effects of heating in historical heritage sites using WSNs to monitor the temperature and humidity conditions during two years.

The design of a WSN for monitoring and conservation of heritage sites depends on the application scenario. It must consider specific requirements, such as the environment, resources, cost, hardware, and system constrains. One of the clues of this kind of WSNs is the routing topology that the messages follow to reach the base station. Several works have been performed to study the configuration of these types of networks [11,12,13]. To face the problem of testing WSNs, different articles have also been found in the literature [14,15]. Therefore, a preliminary analysis about which topology is more adequate should be carried out in this application scenario. This paper provides a performance evaluation of tree and mesh routing topologies of WSNs in a cultural heritage site. The historical heritage site selected was the San Juan Bautista church in Talamanca de Jarama (Madrid, Spain). The tree topology used was the collection tree protocol (CTP) and the mesh topology was the XMESH provided by MEMSIC (Andover, MA, USA). These topologies were selected because they are the most commonly used [16,17,18,19,20,21,22,23]. An experimental study of WSN power efficiency using the XMESH routing topology was reported in [23]. 

The BLIP, the Berkeley Low-power IP stack, and TinyRPL implementations in TinyOS 2.x were used to evaluate the performance of the newly proposed standards and compare them with CTP [24]. Three protocols for WSNs (i.e., CTP, MultiHopLQI and BLIP 6LowPAN) were experimentally compared to assess the performances in terms of latency and packet loss [25]. In this work, CTP exhibited minimum latency and the best reliability. Previous studies compared mesh and tree topologies, but only in a simulated scenario [26,27]. 

To test which routing topology was better for this kind of application, the WSNs were first deployed on the upper floor of the CAEND institute in Arganda del Rey (Madrid, Spain) simulating the church deployment. This deployment is representative of a typical building interior with numerous walls and no direct line of sight between the WSN elements. In the cultural heritage site studied in this work there were almost no fixed obstacles and, therefore, direct line of sight between the WSN elements. Both scenarios had a rather similar arrangement; the motes were distributed in an elongated rectangular surface, forming two parallel lines with the base station to one end. This arrangement is very common in buildings, such as warehouses, churches, offices, etc. Motes were tested point to point and several communication parameters were measured, such as the received signal strength indication (RSSI), the throughput, and the transmission bandwidth. It was found that this network was appropriate for this kind of deployment. We, therefore, decided to carry out a more comprehensive study to determine which topology was preferred in a particular scenario.

## 2. WSN

When a WSN is deployed in a real scenario, many communication problems arise due to the uncontrolled circumstances; for example, crowd intensity, presence of no fixed obstacles, electrical power outages, etc. Each cultural building has unique characteristics from the point of view of design, construction, and materials used to build it. In particular, in the cultural heritage site selected in this work, many communications problems appeared. During the celebration of catholic ceremonies, such as masses, Passion Week, etc. the communications were affected due to the presence of people, sculptures, and large quantities of flowers for the processions. Therefore, it is very important to test this type of network in a real deployment to evaluate which routing protocol is more effective for the required application. Comparing the number of lost messages and the lifetime of the network makes it possible to analyze the network behavior for a particular scenario and to choose the best routing topology for the application.

In this section the wireless sensor system and components developed by the authors are described.

### 2.1. The Hardware Platform

Two WSNs composed of Mica2 motes purchased from MEMSIC (Andover, MA, USA) [28] using different routing topologies, mesh or tree, were employed. The Mica2 motes come in three models according to their RF frequency band: the MPR400 (915 MHz), MPR410 (433 MHz), and MPR420 (315 MHz). The motes use the Chipcon CC1000, FSK modulated radio and are controlled by an Atmega128L micro-controller with a frequency-tunable radio with extended range. The MPR400 model, emitting maximum power, was used in this experiment. Software programs for motes were written in NesC (an object-oriented extension of C) and run using the TinyOS operating system [29]. Both WSNs were composed of seven motes and the base station. Motes were programmed to send a data package of 41 bytes every 2 min, which was enough to monitor the slow temperature changes in the buildings. As mentioned above, this work arose from a project to study the effects of heating in historical heritage sites using WSNs to monitor the temperature and humidity conditions inside and outside the walls during periods of weeks.

### 2.2. Routing Algorithms

The two different routing topologies used in this work, mesh and tree, respectively, using the XMESH and the CTP protocol, are described below.

#### 2.2.1. XMESH

XMESH is a full-featured, multi-hop, ad-hoc, mesh networking protocol developed by MEMSIC (Andover, MA, USA) for wireless networks [28]. An XMESH network consists of nodes (motes) that wirelessly communicate with each other and are capable of hopping radio messages to a base station where they are passed to a PC or other client. The hopping effectively extends the radio communication range and reduces the power required to transmit messages. By hopping data in this way, XMESH can provide two critical benefits: improved radio coverage and improved reliability. Two nodes do not need to be within direct radio range of each other to communicate. A message can be delivered to one or more nodes in between, which will route the data to its final destination. Likewise, if there is a bad radio link between two nodes, that obstacle can be overcome by rerouting around the area of bad service. 

#### 2.2.2. Collection Tree Protocol (CTP)

The TinyOS-2.x operating system provides a well-tested, tree-based routing protocol called the collection tree protocol (CTP). A number of nodes in a network advertise themselves as tree roots. The remaining nodes form a set of routing trees to these roots. CTP is address-free in that a node does not send a packet to a particular root; instead, it implicitly chooses a root by choosing the next hop. Nodes generate routes to roots using a routing gradient [30]. CTP uses expected transmissions (ETX) as its routing gradient, with roots having an ETX of 0. The ETX of a node is the ETX of its parent plus the ETX of its link to its parent. This additive measure assumes that nodes use link-level retransmissions. Given a choice of valid routes, CTP should choose the one with the lowest ETX value. 

The main problems that emerge in a CTP network are routing loops and packet duplication. Routing loops generally occur when a node chooses a new route that has a significantly higher ETX than the older one, perhaps in response to losing connectivity with a candidate parent. If the new route includes a node that was a descendant, then a loop occurs. Packet duplication is an additional problem that can occur in CTP. This occurs when a node receives a data frame successfully and transmits an acknowledgment (ACK), but the ACK is not received. The sender retransmits the packet, and the receiver receives it a second time. This can have disastrous effects over multiple hops, as the duplication is exponential. For example, if each hop, on average, produces one duplicate, then on the first hop there will be two packets, on the second hop there will be four, on the third hop there will be eight, and so on.

## 3. Scenario of the WSN Deployment

**Figure 1:**
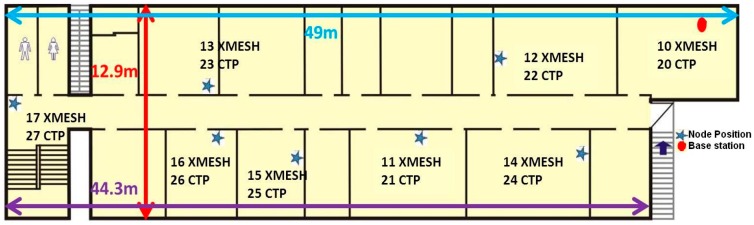
WSN deployment on an upper floor of the CAEND institute.

The historical heritage site selected was San Juan Bautista church in Talamanca de Jarama (Madrid, Spain). As mentioned in Section 1, to test which routing topology was better for this kind of application, the WSNs were first deployed on the upper floor of the CAEND institute in Arganda del Rey (Madrid, Spain) (Figure 1), simulating the church deployment. In this location there was no direct line of sight between the WSN elements and walls are made of bricks and glass. The first WSN was composed of seven motes (11–17) and the base station (10) using the XMESH protocol. The second WSN was also composed of seven motes (21–27) and the base station (20) using the CTP protocol.

**Figure 2:**
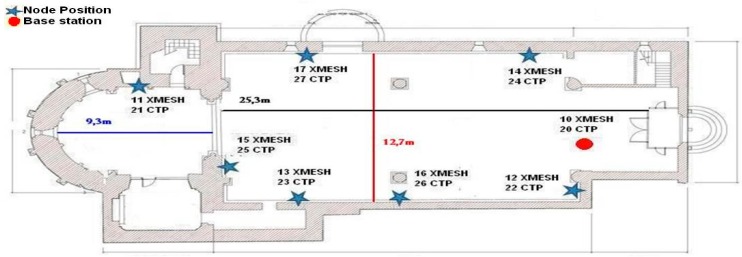
WSN deployment in the church of Talamanca de Jarama.

Since both WSNs worked well at the institute, they were tested in San Juan Bautista church as depicted in Figure 2.

## 4. Performance Evaluation

As previously mentioned, two parameters were selected to evaluate the performance of the routing topologies of WSNs, the percentage of received messages and the lifetime of the network. To analyze in more detail which topology gave the best performance, other communication parameters were measured, such as the number of hops required for every message to reach the base station, the parent identifier for each message, and the Received Signal Strength indicator (RSSI) values for each mote. 

To compute the number of lost messages, messages were labeled with consecutive identification numbers and the missing numbers were spotted and summed. Representing this number of lost messages as a function of time, the behavior of the WSN can be analyzed and malfunctions can be detected. 

Energy efficiency and optimal performance are essential for WSNs that operate in remote environments with difficult access. Energy efficiency can be improved with an appropriate choice of the WSN topology and communication protocols, with the energy required for communication scaling with distance (d) in a d2 to d4 relation. Therefore, the lifetime of the WSN has been studied as an indicator of energy efficiency. Some computer simulations have been performed to study the energy efficiency using different topologies [27], and different assumptions about the radio parameters in transmit and receive modes also change the relative advantages of different topology and routing protocols.

The performance of the WSN was also monitored using additional parameters, related to the routing of messages within the WSN, these parameters were the number of hops, the parent identifier, and the RSSI values.

The number of hops is the number of times a packet travels from the source through the intermediate nodes to reach the base station. A data point with one hop indicates that the message was received directly by the base station, two hops means that there was one intermediate mote, and so on.

The parent identifier is the identifier of the first mote that receives the message. If the base station has a direct connection to the mote sending the message, then the base station is identified as the parent.

The RSSI values are measured for each mote in every position and these values can be related with the parent identifier of each mote. 

Henceforth, we use the expression “crowd intensity” to mean the number of people walking in the vicinity of the WSN, leading to signal interference. Crowd intensity was higher during working days from Monday to Friday, and lower during weekends and holiday periods. Three different experiments were carried out to evaluate the selected routing topologies: Experiments 1 and 2 were performed at the CAEND institute with high and low crowd intensity, respectively, and with no direct line of sight between motes. To check the robustness of the results obtained, the motes were exchanged but keeping the same general spatial configuration. Experiment 3 was performed at the church deployment with occasional high crowd intensities during masses and with direct line of sight between motes. 

## 5. Results

**Figure 3:**
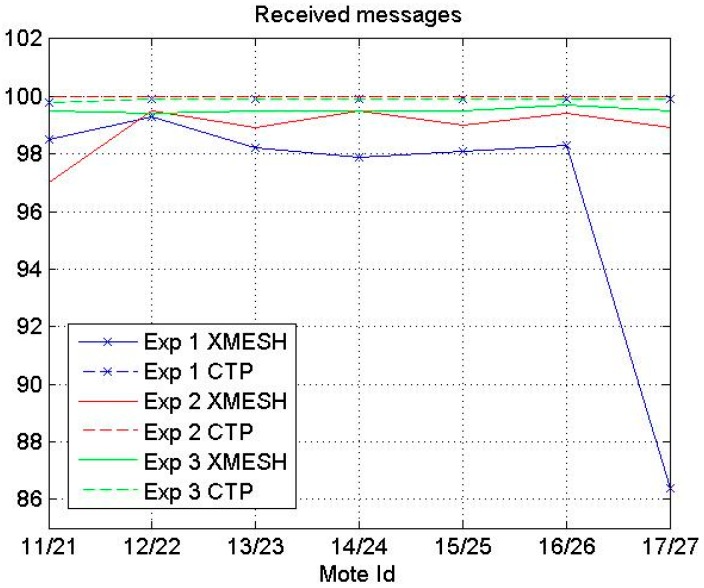
Percentage of received messages using the XMESH and CTP protocols.

In this section, the results obtained with the WSNs during the three experiments are presented. The number of lost messages was compared with the lifetime of the network to analyze the network behavior for a particular scenario and to detect hardware failures. To prevent erroneous data due to the lack of battery power needed to compute the number of lost messages, only measurements up to 8400 min were considered. Figure 3 presents the percentages of received messages using the XMESH and CTP protocols in the three experiments for each mote. The percentage of received messages using the XMESH protocol was always lower compared to the CTP protocol. In Experiment 1 the difference between the average percentages of received messages using both protocols was the largest, probably due to the high crowd intensity during the test. In Experiment 3 this difference was the smallest since there was direct line of sight between motes. 

The nodes closest to the base station (Motes 12 and 14) in Experiment 2 were the motes with the lowest number of lost messages using the XMESH protocol, but this tendency was not observed when the CTP protocol was used in the same experiment.

**Figure 4:**
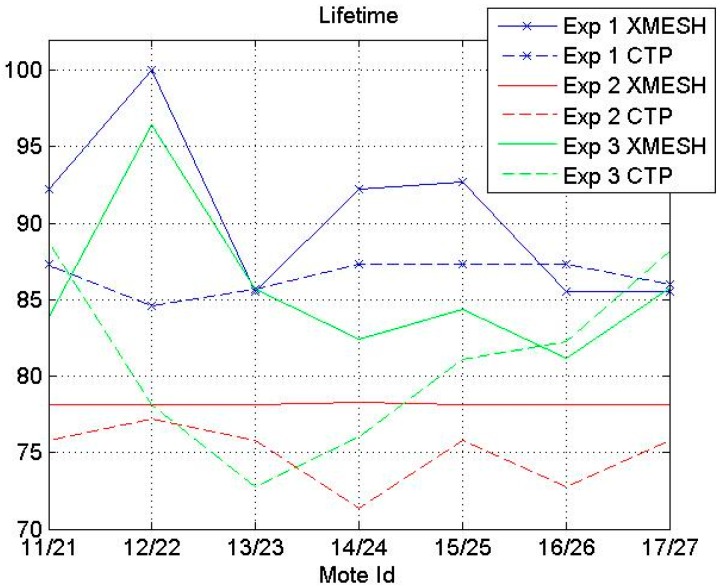
Percentage of the relative lifetime of each mote using the XMESH and CTP protocols.

The percentage of the relative lifetime of each mote using the XMESH and the CTP protocols in the experiments is presented in Figure 4. The relative lifetime for each experiment means the lifetime of each mote considering 100% the lifetime of the last mote depleting its energy. The value of 100% corresponds to 13,384 min. On average the WSN using the CTP protocol depleted its energy faster than the WSN using the XMESH protocol in all experiments. Experiment 1 had the longest lifetime and Experiment 2 the shortest one. In Experiment 1 the results obtained using the XMESH protocol had more dispersion than using the CTP protocol, while the reverse situation was observed in Experiments 2 and 3. Figure 4 also shows that, in general, the motes located at longer distances from the base station stopped emitting before those closer to the base station.

**Figure 5:**
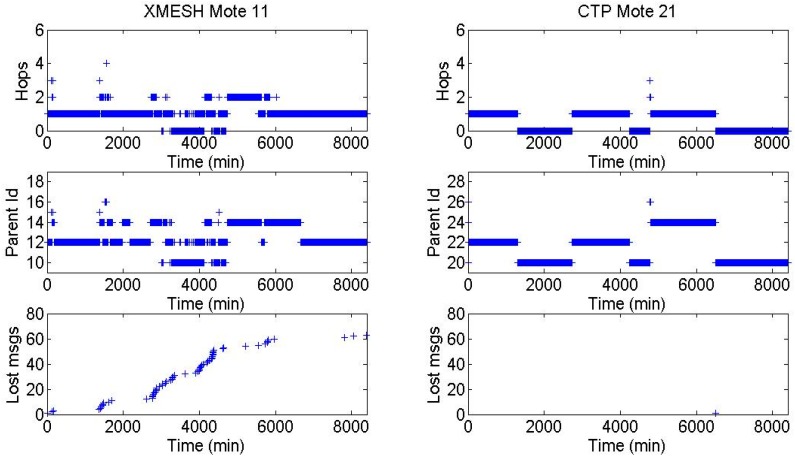
Number of hops, parent identifiers of each message, and total number of lost messages as a function of time using the XMESH (Mote 11) and CTP (Mote 21) protocols in Experiment 1.

In Experiment 3, since there was direct line of sight, both WSNs worked well and were very stable, and almost all motes had a direct connection to the base station. Therefore, a more detailed study was developed specifically for Experiments 1 and 2 to understand why the CTP protocol performed better than the XMESH one. The number of hops, the parent identifiers of each message, and the total number of lost messages as a function of time, using the XMESH (Mote 11) and CTP (Mote 21) protocols during Experiment 1, is shown in Figure 5. This representation illustrates the differences between protocols for Motes in the same positions. It was found that fewer messages were lost using the CTP and the WSN was more stable, i.e., there were fewer changes from one parent to another. Once a parent was selected, it was kept for a longer time interval.

**Figure 6:**
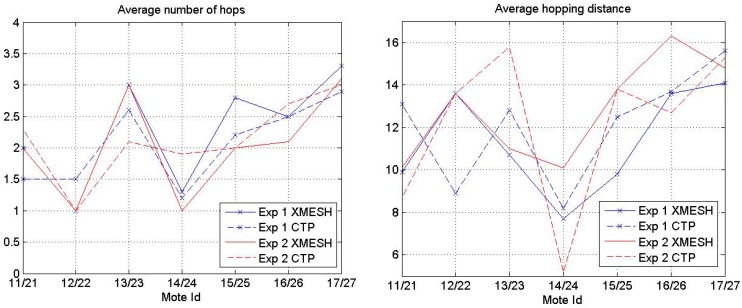
Average number of hops (**left**) and average hopping distance (**right**) of each mote using the XMESH and CTP protocols.

Figure 6 presents the average number of hops made for a message to reach the base station and the average hopping distance reached by each mote (i.e., the distance divided by the average number of hops) using each protocol in Experiments 1 and 2. As expected, the nodes closer to the base station had on average the lowest number of hops for both protocols. In Experiment 1, the average number of hops was always lower (and, therefore, the average hopping distance was larger) for the CTP protocol than for the XMESH protocol, except for Mote 12. In Experiment 2, the average number of hops was, in general, lower (and, therefore, the average hopping distance was larger) for the XMESH protocol than for the CTP protocol. The average number of hops in Experiment 1 was larger than in Experiment 2 due to the higher presence of people.

**Figure 7:**
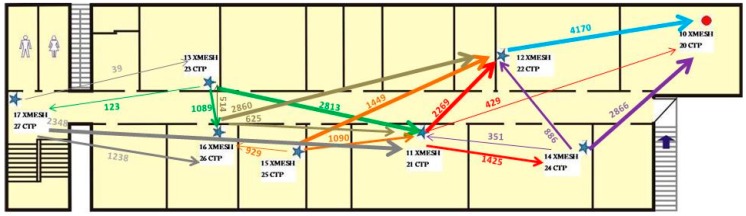
Route distribution for each mote using the XMESH protocol in Experiment 1.

**Figure 8:**
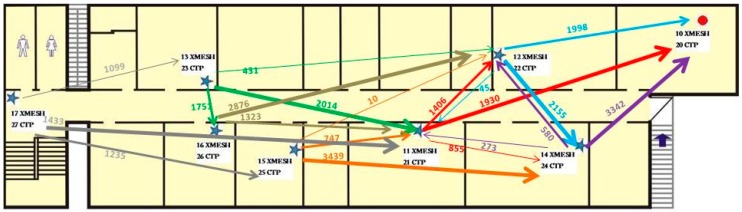
Route distribution for each mote using the CTP protocol in Experiment 1.

The route distribution for each mote using the XMESH and CTP protocols in Experiment 1 is presented in Figure 7 and Figure 8, respectively. For each mote, the three parent identifiers that received the largest number of messages are shown. This type of diagram shows that using the CTP protocol, the network is distributed more efficiently than using the XMESH protocol, since the number of messages received by each mote was more balanced.

The RSSI values of the WSN located at the CAEND institute are shown in Table 1. It should be considered that walls are made of bricks and glass. Mote 22 had the lowest RSSI value with the base station (Mote 20). The best connections were found between Motes 23 and 26 and between 23 and 25.

## 6. Discussion

In this section, a comparison between the performance of the XMESH and CTP protocols tested at the CAEND institute (Experiments 1 and 2) is presented. Two parameters, the percentage of received messages and the lifetime of the WSN, are proposed in this work to analyze which routing protocol is better in a particular deployment. According to the results reported in Figure 3 and Figure 4, the percentage of received messages using the XMESH protocol was always lower than that using the CTP protocol. In general, the WSN using the CTP protocol depleted its energy faster. 

A more detailed analysis of the data was developed by us to understand why the WSN using the CTP protocol performed better, but depleted its energy faster, than the WSN using the XMESH protocol in both experiments. For that purpose, the communication parameters described above will be presented as a function of time during the experiment.

**Figure 9:**
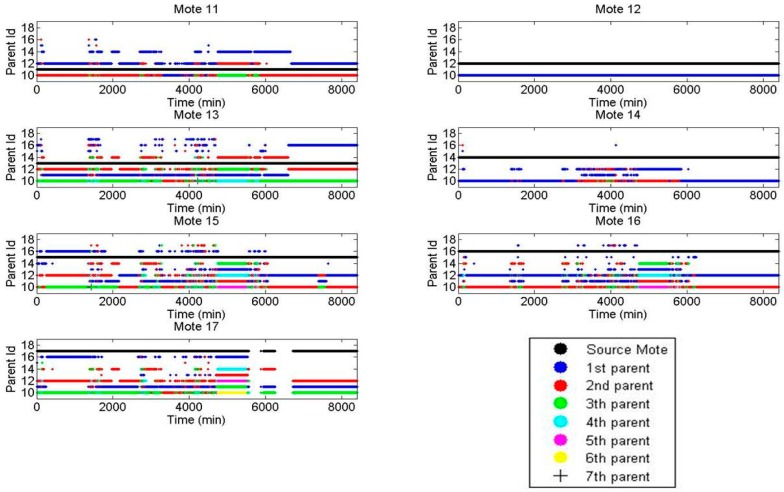
Route distribution for each mote using the XMESH protocol in Experiment 1.

**Figure 10:**
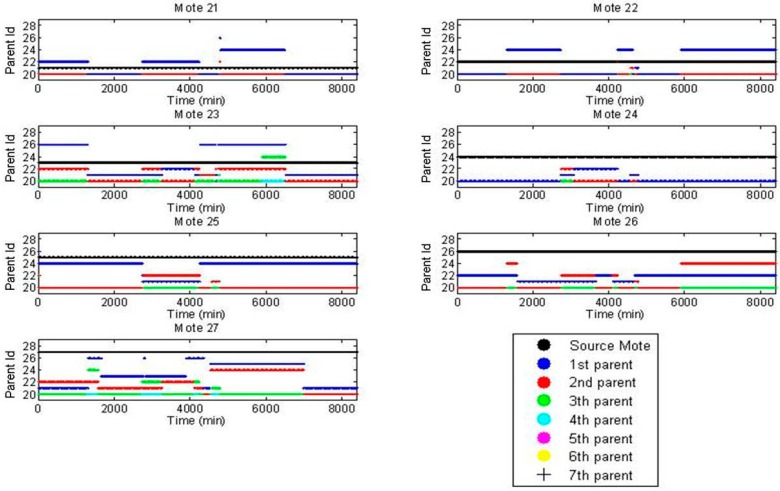
Route distribution for each mote using the CTP protocol in Experiment 1.

For each mote, the route followed by each message as a function of time is shown in Figure 9 and Figure 10 using the XMESH and CTP protocols in Experiment 1, respectively.

The black dot represents the mote that created the message (source mote), a blue dot represents the parent of a mote (the first parent), a red dot the parent of the parent (the second parent, i.e., a grandparent in human terms), a green dot the third parent, a cyan dot the fourth parent, a magenta dot the fifth parent, a yellow dot the sixth parent, and a black cross the seventh parent. It can be seen that the CTP network was more stable, i.e., there were fewer changes from one parent to another. Indeed, once a parent is selected it is kept for a longer time interval (which is visualized as a continuous line of the same color). Since this experiment starts on Monday morning, it can be observed that, during the weekend, the XMESH configuration was more stable than during working days, but compared with CTP the number of hops was generally larger.

**Figure 11:**
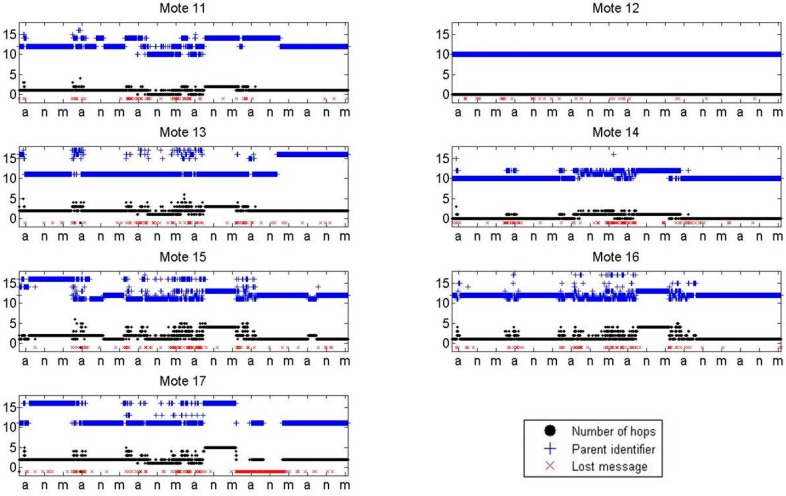
Lost messages (red color), number of hops made for a message to reach the base station (black color), and parent identifiers (blue color) with respect to time for each mote. Data corresponds to the XMESH protocol in Experiment 1. In the x-axis, “m” means morning, “a” is afternoon, and “n” is night.

The lost messages (red color), number of hops made for a message to reach the base station (black color), and parent identifiers (blue color) as a function of time for each mote using the XMESH protocol, are shown in Figure 11. In the x-axis the time interval is presented as a day period, morning (m) from 7:00 to 15:00, afternoon (a) from 15:00 to 23:00, and night (n) from 23:00 to 7:00. The data considered in Figure 11 starts on Monday morning and lasts until Sunday morning. When a lost message occurs, a red cross is plotted with a value of −1. This representation of the data serves to point out possible relationships between the lost messages and the change of the parent identifier or the number of hops required to reach the base station. It can be observed that, in many cases, a message loss using the XMESH protocol is associated with a route change. Mote 12 had the lowest number of lost messages; it did not change its parent.

With this representation, it can also be seen that, commonly, the received message losses occurred in the morning, probably due to the higher crowd intensity. During the weekend, when the crowd intensity was the lowest, fewer messages were lost and the route was more stable since the motes almost did not change the parent identifier.

**Figure 12:**
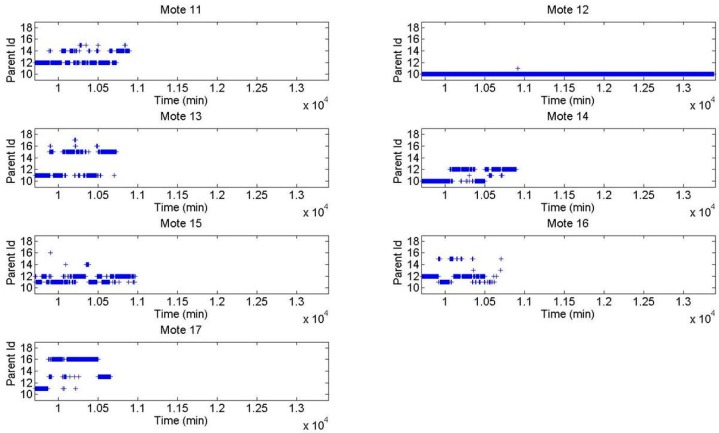
Parent identifiers of each message using the XMESH protocol during the last period of the WSN lifetime in Experiment 1.

**Figure 13:**
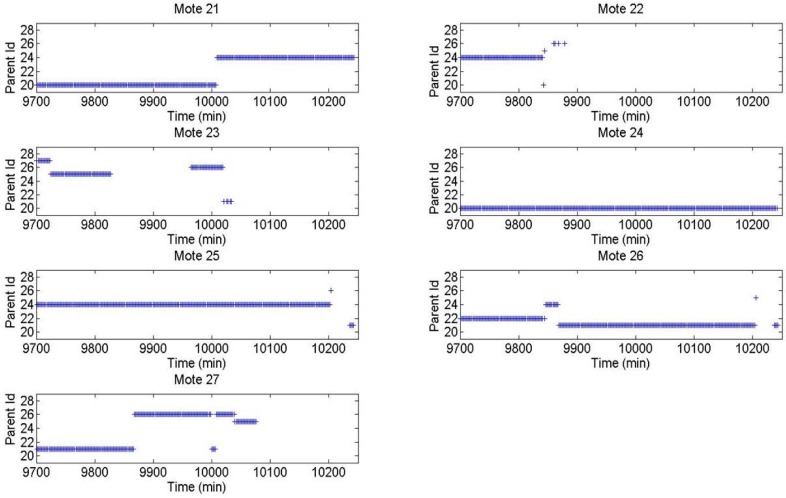
Parent identifiers of each message using the CTP protocol during the last period of the WSN lifetime in Experiment 1.

The analysis of the parent identifiers for each message during the last period of the WSN lifetime (displayed in Figure 12 and Figure 13 for the XMESH and CTP protocols, respectively) provides more insight about the network behavior. The data shown starts when the first nodes disconnected from the WSN and ends when the last nodes disconnected. A detailed analysis was performed when multiple nodes simultaneously disconnected from the network, trying to understand if they depleted their energy or if they died because their parents died in advance.

In the case of the XMESH protocol, Motes 13, 16, and 17 disconnected first from the network. Motes 13 and 16 depleted their energy since their parents were still connected to the WSN. Therefore, Mote 17 probably died because it did not have enough energy to connect with more distant nodes. Then, Motes 11 and 14 depleted their energy since they could have otherwise connected with Mote 12. After that, Mote 15 depleted its energy, because its parent was still alive. Finally, Mote 12 lasted a longer time and, therefore, it was the final parent of all motes.

For the CTP protocol, Motes 22, 23, and 27 depleted their energy first, in this order. Then, Motes 21, 25, and 26 died due to the cascade effect created by their parent’s death. Finally, Mote 24 lasted a longer time and was, therefore, the final parent of all motes. 

As previously mentioned, this experiment was repeated to check the robustness of the results obtained, the motes were exchanged but keeping the same general spatial configuration (Experiment 2). In this second experiment, similar results were obtained.

Several conclusions can be drawn from the detailed analysis of Experiments 1 and 2. The CTP network performed better than the XMESH network in all the experiments, meaning that fewer messages were lost. The main reason for this behavior is that the XMESH network tends to reconfigure itself faster than the CTP network when there are communication problems, mainly caused by the movement of people in the real deployment. If the location of the network is static, this is a disadvantage because the time required to reconfigure the network can lead to message loss. The CTP algorithm is much more stable when reconfiguring the network structure, thus producing a smaller number of lost messages. In the almost absence of people interfering with the radio signal, during the evenings and weekends, as well as in Experiment 2, it was found that the behavior of both networks, in terms of its configuration, was very similar and the number of lost messages was relatively low. Another aspect related to the stability of the WSN is that, in the XMESH network, the percentage of lost messages increases with the number of hops given by a message from the source to the base station. It was also observed that the average hopping distance reached by each mote did not affect the message loss.

It is well known that batteries discharge more quickly in cold weather than in hot weather conditions. This might explain why the energy efficiency corresponding to Experiment 1, performed in late May, was high compared to Experiment 2, performed in January. 

Finally, in all experiments, it was found that, on average, the WSN using the CTP protocol depleted its energy faster than the one using the XMESH protocol. In both protocols, nodes closer to the base station, handling more traffic, lasted longer than the other nodes. Therefore, the number of transmissions does not affect the lifetime of the motes.

## 7. Conclusions 

This paper provides a performance evaluation of tree and mesh routing topologies of wireless sensor networks (WSNs) in San Juan Bautista church in Talamanca de Jarama (Madrid, Spain). To test which routing topology was better for this kind of application, the WSNs were first deployed on the upper floor of the CAEND institute in Arganda del Rey (Madrid, Spain) simulating the church deployment. Two parameters were selected to evaluate the performance of the routing topologies selected, the percentage of received messages and the lifetime of the network. To analyze in more detail which topology gave the best performance, other communication parameters were measured, such as the number of hops required for every message to reach the base station, the parent identifier for each message, and the RSSI values for each mote. 

The tree topology used was the collection tree protocol and the mesh topology was the XMESH provided by MEMSIC (Andover, MA, USA). These topologies were selected because they are the most commonly used.

Three different experiments were carried out to evaluate the selected routing topologies. Experiments 1 and 2 were conducted at the CAEND institute with high and low crowd intensity, respectively, while Experiment 3 was performed at the church deployment with occasional high crowd intensities during mass times.

In this study, the following conclusions were obtained:

- The CTP network performed better than the XMESH network in all experiments, meaning that fewer messages were lost. The main reason of this behavior is that the XMESH network tends to reconfigure itself faster than the CTP one, if there are communication problems, which were mainly due to the movement of people. This could be an advantage in some applications, but the reverse situation occurs if the location of the network is static, mainly because the time required to reconfigure the network can lead to message loss. When XMESH became more unstable, mainly due to high crowd intensity, the number of hops increased and message loss was associated with route changes.
- The CTP network was more stable, i.e., there were fewer changes from one parent to another.
- People presence affected the XMESH network more than the CTP one. Under quiet conditions (low people presence), during evenings and weekends, as well as in Experiment 2 performed during a holiday period, it was found that the behavior of both networks, in terms of network configuration, was very similar and the number of lost messages was reduced.

In all experiments, it was found that, on average, the WSN using the CTP protocol depleted its energy faster than the WSN using the XMESH protocol. In both protocols, nodes closer to the base station handling more traffic lasted longer than the other nodes. Therefore, the number of transmissions does not affect the lifetime of the motes.

The conclusions of this work are constrained to WSNs using the very common CTP and XMESH protocols, as representative of tree and mesh topologies, respectively. Future work using different protocols is needed to verify if our findings apply to tree and mesh topologies in general. This task is out of the scope of the present work, which is one of the first attempts to provide an experimental evaluation to test the performance of WSNs.

## Figures and Tables

**Table 1:** RSSI values of the WSN at the CAEND institute.

RSSI	Mote 20	Mote 21	Mote 22	Mote 23	Mote 24	Mote 25	Mote 26	Mote 27
Mote 21	244	X	38	230	190	125	215	260
Mote 22	182	138	X	265	169	195	248	X
Mote 23	X	230	265	X	X	76	35	220
Mote 24	193	190	169	X	X	252	X	X
Mote 25	X	125	195	76	252	X	146	291
Mote 26	X	215	248	35	X	146	X	209
Mote 27	X	260	X	220	X	291	209	X
